# Early Functional Treatment and Modern Cast Making for Indications in Hand Surgery

**DOI:** 10.1155/2016/5726979

**Published:** 2016-04-17

**Authors:** S. Bohr, N. Pallua

**Affiliations:** ^1^Department of Plastic and Hand Surgery, Burn Center, University Clinics, RWTH Aachen University, Pauwelsstrasse 30, 9th Floor, B2 R11, 52074 Aachen, Germany; ^2^Center for Engineering in Medicine/Surgical Services, Massachusetts General Hospital, Harvard Medical School and Shriners Hospitals for Children, Boston, MA 02114, USA; ^3^Department of Trauma Surgery, Plastic and Reconstructive Surgery, University Clinics, Goettingen, Germany

## Abstract

Cast treatment can serve both as a nonsurgical treatment option and as a means for providing postoperative protection. However, with the duration of immobilization intervals, the benefits of cast treatment, especially in hand surgery, are at risk of being outweighed by undesired drawbacks such as joint stiffening and contracture formation. In order to minimize potential complications commonly associated with cast treatment, efforts to further improve cast making must attempt to reconcile two conflicting objectives: (1) to achieve stability and rigidity at the site of injury (e.g., fracture retention) and (2) to allow free range of joint movement as early as possible. In addition, in order to assure patient compliance, modern cast treatments should aim to improve wearing-comfort of the cast. This paper describes modern cast designs for four common types hand injuries, with sample cases highlighting the clinical outcome of each treatment.

## 1. Introduction

Cast treatment not only is well established as a nonsurgical treatment option for a variety of posttraumatic conditions of the hand but also is an indispensable tool for providing postoperative protection, for example, for reduced fractures, reconstructed ligaments, tendons, or nerves. Immobilization, however, also has well-known drawbacks which can negatively impact the time of return to “normal hand activity” and the overall clinical outcome as a whole [1]. Common sequela of prolonged cast immobilization includes arthrofibrosis, inflammatory tenodesis, or contracture formation [2]. Efforts to further improve cast making—with regard to both cast materials and cast making techniques—must therefore attempt to reconcile two conflicting objectives: (1) to achieve stability and rigidity at the site of injury (e.g., fracture retention) and (2) to allow free range of motion as early as possible following injury. Modern cast designs do not only have the potential for minimizing the drawbacks of immobilization but can also improve compliance, as cast treatment is much more likely to be accepted by patients if emphasis is put on light materials, wearing-comfort, and minimizing restriction of movement with the goal of allowing normal daily activity [3].

The cast designs presented in this paper reflect the consistent use of modern cast materials and the consistent application of the* early functional treatment* principle first proposed by Bunnell [4], Boehler [5], Kleinert et al. [6], and others as early as in the 1940s. The authors of this paper believe that the presented cast designs can both improve overall clinical outcomes and reduce the socioeconomic cost of injury by speeding up recovery and allowing an earlier return to the workplace.

## 2. Key Considerations and Cast Making Technique

### 2.1. Key Therapeutic Considerations

In this paper, we refer to the presented cast designs as* orthoses*. They can be applied following hand surgery or as a treatment option for closed fracture reduction as soon as primary wound-healing or soft-tissue swelling permits, usually within a week following the initial trauma. In the interim, traditional plaster casts typically serve as a valuable tool for reducing pain or for temporarily stabilizing fractures. Before using one of the presented cast designs, a key therapeutic decision needs to be made about whether the prescribed orthoses should be* wrist-free* or not.

### 2.2. Key Anatomical Considerations

Since orthoses are* skin-tight*, it is necessary to consider typical anatomical landmarks with a heightened risk of pressure ulcers such as the* styloid process of radius *or* ulna*. The well-established principles of immobilization in either a* functional* (i.e., a balanced passive extensor and flexor muscle tone [7]) or* intrinsic-plus* position of the hand apply as usual. Among hand surgeons, the* intrinsic-plus* position of the hand is widely accepted to be best suited for immobilization, with the wrist extended 30°, the MCPJ flexed > 80°, and the PIPJ and the DIPJ fully extended [4].

### 2.3. Key Technical Considerations

**Figure 1:**
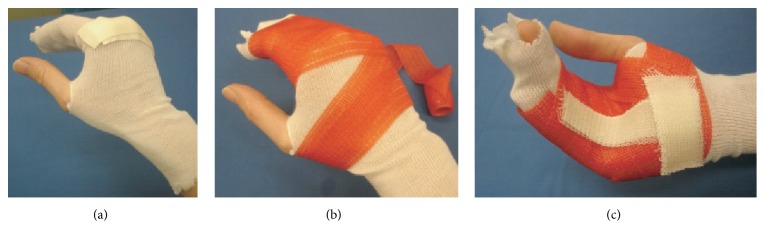
Application of custom-made orthoses. (a) Basic cushioning with tube-gauze and* Microfoam™*-tape; function: (i) cushioning and (ii) absorption of skin moisture. (b) Circular application of semirigid cast role (red: Soft-Cast*™*, width 2.5–5 cm). No more than two to four layers of the material are necessary. (c) Integration of rigid cast stripes (white: Primacast*™*). There is sufficient time to bring each joint and finger in the desired functional position. Note: hardening through polymerization is strongly accelerated (<3 min) by the application of water* via* a moist bandage. A final trim of semirigid cast material (in contrast to rigid cast material) can be easily performed after hardening. If out-of-cast physiotherapy is desired, the orthoses can be cut open dorsally and self-adhesive Velcro*™*-tape applied to refitting as seen in Figure 2. Note: disposable rubber gloves should be worn to avoid chronic exposure to solvent in the synthetic cast material.

The main structure of the orthoses presented in this paper consists of two to four layers of semirigid or flexible polymerized synthetic fabric (e.g.,* Soft-Cast*). The required stability and rigidity at the desired anatomical location (e.g., the fracture-zone) are achieved through two different cast techniques used in the design of each of the presented orthoses. The* circular* cast technique relies on the laws of hydrostatics (see also Discussion), whereby pressure applied to the inner surface of the orthoses (through soft-tissue interaction) will increase overall rigidity of the orthoses. The* linear* cast technique relies on additional layers of rigid synthetics (e.g.,* Primacast* as illustrated by Figure 1) diverting undesired lever-arm force form the sight of injury [7–9].

### 2.4. Materials Used

The following materials used for cast making in the sample cases presented in this paper can be substituted with a variety of comparable products:

1. Tube-gauze (different sizes): tg (tube-gauze)®-Lohmann & Rauscher GmbH & Co. KG.
2. Localized cushioning: Microfoam-3M/Johnson & Johnson.
3. Semirigid cast: Soft-Cast-3M/Johnson & Johnson.
4. Rigid cast: Primacast-3M/Johnson & Johnson.
5. Velcro (self-adhesive tape): Hakupa*™*-Otto Bock.
6. Scotch tape for rim-cushioning: Hakupa-Otto Bock.

### 2.5. Step-by-Step Application of Custom-Made Orthoses

The principle steps in the application of custom-made orthoses are depicted in Figure 1.

## 3. Sample Cases

**Figure 2:**
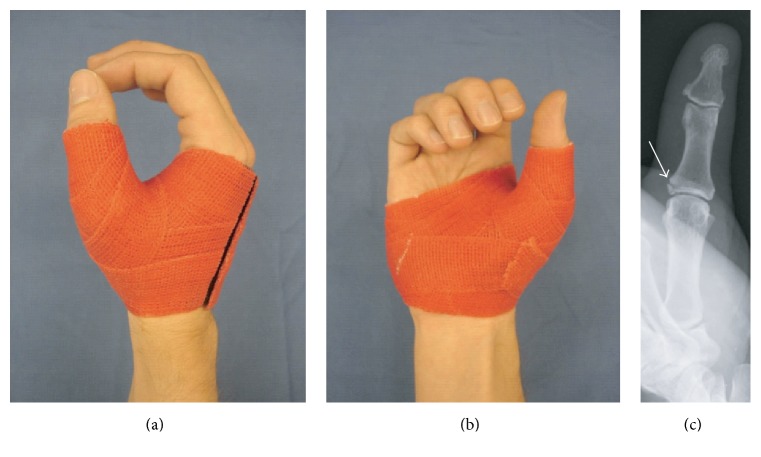
Thumb orthoses: (a) dorsoradial and (b) palmar view and (c) case sample: X-ray at 6 weeks after wearing orthoses following bony ulnar collateral ligament traumatic lesion (DASH analysis of case series: *n* = 11; score-mean = 29.9 ± 1.73 SD; DASH follow-up interval (weeks): 9.5 ± 2.01 SD).

**Figure 3:**
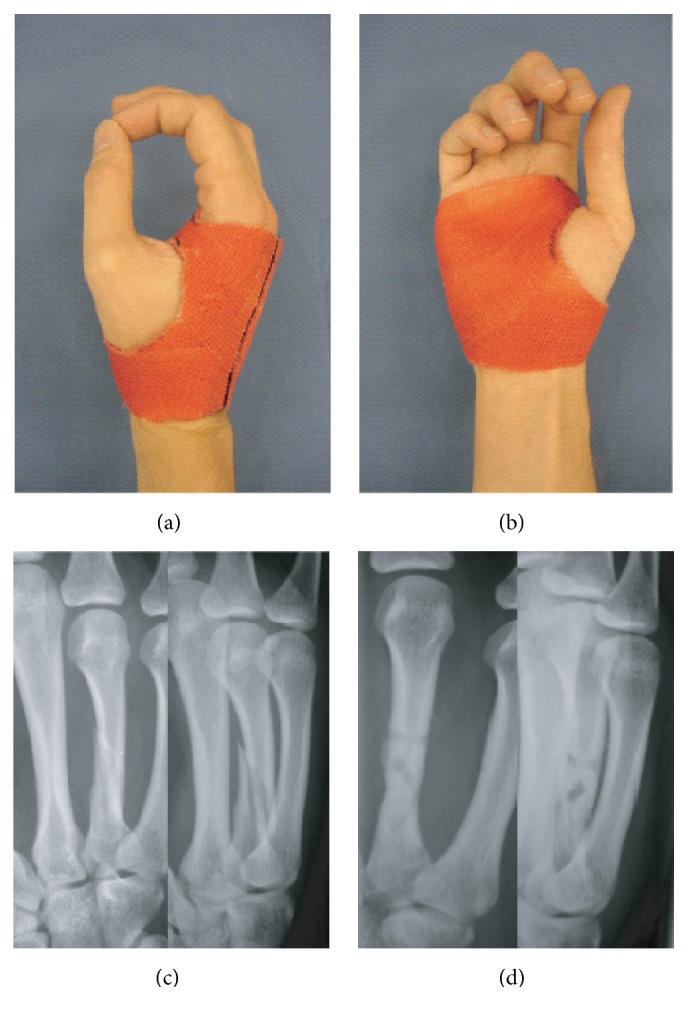
Middlehand orthoses (fingers excluded): (a) dorsoradial and (b) palmar view; case sample: displaced 4th metacarpal fracture, (c) X-ray on admission and (d) at 6 weeks following open reduction and fixation (resorbable implant; 2 mm LactoSorb® plate) (DASH analysis of case series: *n* = 15; score-mean = 32.07 ± 3.54 SD; DASH follow-up interval (weeks): 11.57 ± 0.77 SD).

**Figure 4:**
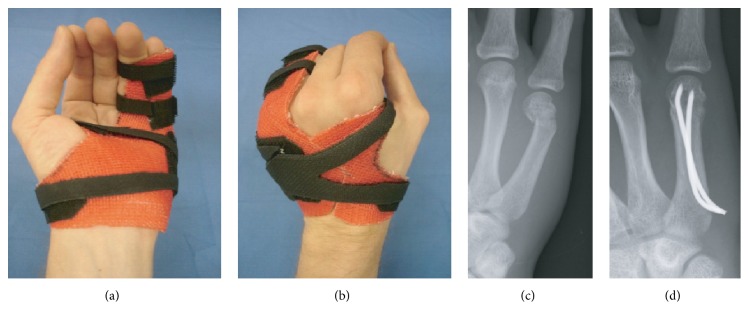
Middlehand orthoses (fingers included): (a) palmar and (b) dorsoradial view; case sample: angulated fracture of the 5th metacarpal head, (c) X-ray on admission and (d) at 6 weeks following anterograde nailing technique (2x intramedullary 2 mm k-wire) (DASH analysis of case series: *n* = 17; score-mean = 32.37 ± 2.53 SD; DASH follow-up interval (weeks): 11.68 ± 1.17 SD).

**Figure 5:**
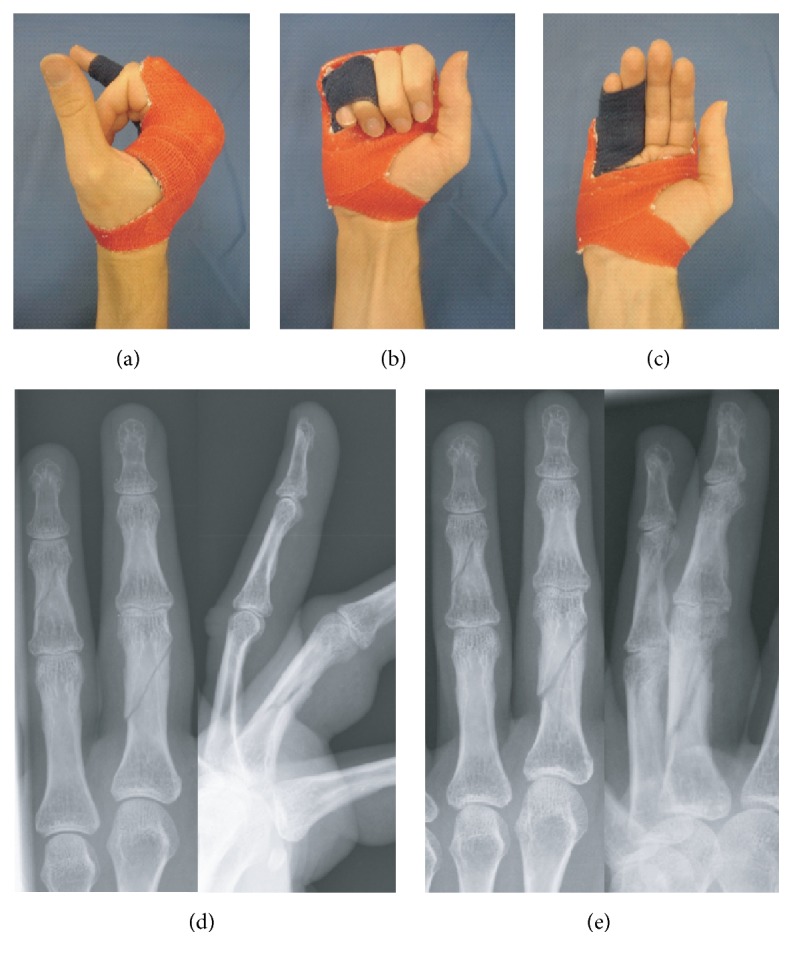
Dynamic finger orthoses with taping: (a) radial, (b) dorsoradial, and (c) palmar view; case sample: nondisplaced 4th proximal phalangeal/5th middle phalangeal fracture, (d) X-ray on admission and (e) at 5 weeks (DASH analysis of case series: *n* = 27; score-mean = 33.30 ± 9.99 SD; DASH follow-up interval (weeks): 9.08 ± 2.33 SD).

The purpose of this paper is to describe designs and indications of modern cast making in hand surgery which keep immobilization to a minimum and are consistent with the principle of* early functional treatment*. The choice of cast treatment is only one of a number of prognostic factors that determine the overall clinical outcome in a particular case [7], with the type and severity of injury likely the most important factor. It is therefore difficult to support the effectiveness of a particular form of cast treatment with authoritative statistical evidence. However, the authors of this paper have attempted to evaluate the effectiveness of the different orthoses described in this paper through the use of* Disabilities of the Arm, Shoulder, and Hand* (DASH) [10] follow-up questionnaires for a series of cases similar to each of the sample cases presented below. DASH scores represent a highly standardized and validated tool designed to measure physical function and symptoms in connection with musculoskeletal disorders of the upper limb in patients in a self-administered format. For the purpose of this study, a single endpoint usually within 4 weeks following the end of cast treatment was performed. DASH questionnaires generate a score on a scale between zero and 100, where a high score represents a poor clinical outcome as follows: <24 = minimal difficulty/excellent; 25–49 = mild difficulty/good; 50–74 = considerable difficulty/fair; and >75 = severe difficulty/poor-disabled. The mean DASH scores are included in the captions of the figures included for each sample case/cast design below (see Figures 2–5).

Different indications will necessitate variations to the design of orthoses, but these variations will not change the key considerations or cast techniques described in this paper.  Table 1 summarizes four common indications for cast treatment of hand injuries and the related cast designs described in this paper.

### 3.1. Thumb Orthoses

*Sample Case*. Nondislocated bony avulsion of the ulnar or radial collateral ligament of the thumb usually can be treated without performing surgery [11] (Figure 2).

### 3.2. Middle Hand Orthoses

*(a) Fingers Excluded*

*Sample Case*. Dislocated metacarpal shaft fractures are typically treated with open reduction procedure and lag screw, titanium, or resorbable poly(l-lactide) plate fixation. The risk of secondary loss or reduction is higher with biodegradable internal fixation compared to conventional titanium implants [12] (Figure 3).

*(b) Fingers Included*

*Sample Case*. A* Boxer's fracture* of the fifth metacarpal commonly indicates closed reduction with k-wire retention and concomitant cast treatment as previously described [13, 14] (Figure 4).

### 3.3. Dynamic Finger Orthoses

*Sample Case*. This variation of custom-made orthoses is specifically designed for the early functional treatment of proximal phalangeal fractures with or without (Figure 5) a surgical reduction procedure. The presented cast design is based on the so-called* double finger stall*, dynamic splinting technique, with the injured finger splinted to its neighbour [15]. This form of “dynamic fracture mobilization” relies on the stabilizing influence exerted by the* extensor tendon sheath apparatus*, preferably with the MCPJ flexed >80°.

## 4. Discussion

For purposes of the four indications described above, treatment was considered successful if no complications attributable to insufficient immobilization occurred, such as secondary loss of fracture-reduction, rotational finger deformity, pseudoarthrosis, or chronic joint-instability with or without pain. Excellent early functional outcomes at the end of cast treatment are reflected in mean DASH-scores below 44 for the relevant series of similar cases for each of the sample cases presented in this paper (see captions of Figures 2–4).

Although the traditional plaster cast ([CaSO_4_(H_2_O)_2_]) remains a well-accepted treatment option for certain indications—especially in the immediate posttraumatic interval when soft-tissue swelling and primary wound healing occur [16]—it lacks certain key characteristics required for modern cast making. The introduction of synthetic cast material [17, 18] required a reevaluation of previously well-established cast making principles and the introduction of new techniques. Sarmiento and Latta, for example, applied* Pascal's law* of hydrostatics in developing his* bracing* technique, which avoids joint-immobilization in connection with the treatment of long bone fractures (especially the* tibia*) without compromising fracture retention and stability [19]. The* bracing* technique uses the soft tissue surrounding bone to stabilize a fracture site by preventing it from changing volume by means of a circular cast. Additional lessons for achieving the required level of stability while minimizing immobilization of adjacent joints can be drawn from the* three-point-pressure* principle in fracture retention, which was first widely promoted by Charnley* in the 1940s* [20].

Immobilization by means of cast treatment remains a well-established treatment option for a variety of posttraumatic conditions of the hand [21, 22]. However, it is important to be mindful of the potential drawbacks commonly associated with immobilization. There is strong empirical data which suggests that overall clinical outcomes can be improved through treatment regimes that are commonly referred to as* early functional treatment* and that are designed to (i) reduce the time of immobilization and (ii) minimize the restriction of active or passive joint movement [23, 24]. Modern cast treatments for hand injuries, like the cast making technique presented in this paper, must therefore attempt to achieve the benefits associated with early functional treatment without sacrificing the fundamental (but often conflicting) objective of ensuring stability and rigidity at the site of injury.

The utility of the cast making technique presented in this paper is not limited to the four sample cases presented in this paper but can be adapted to a great variety of clinical settings using a modular approach (Table 1). In addition, in the majority of clinical settings, we found no reason to restrict the use of the presented orthoses in children if aged above six years. Further advantages of the presented cast making technique we have observed include (i) a considerable reduction in weight (65–80 g) and bulkiness, (ii) radiolucency which permits in-cast X-ray examination, (iii) overall improved patient comfort and compliance, and (iv) an early rehabilitation and reintegration of the patient into the workplace. We further found that the orthoses described in this paper can be applied by qualified nursing staff with limited additional training, costs for materials of approximately 7–9$/€ per orthoses, and a time expenditure of less than 20 minutes. This makes the presented orthoses preferable over solutions offered by orthotists or commercially available orthoses.

## Figures and Tables

**Table 1:** 

Type of cast	Variant	Site of lesion/indications	Treatment specifications
Thumb orthoses (Figure 2)	DIPJ (excluded or included)	Sprained thumb, IPJ & MCPJ instability (L), 1st metacarpal & proximal phalangeal fracture (F and FC), osteoarthritis (D)	Up to 8 weeks; definite treatment or postoperative protection IPJ & MCPJ

Middlehand orthoses	Fingers excluded (Figure 3)	Different types of metacarpal fractures (F and FC)	Four–six weeks; non/postoperatively
	Fingers included (Figure 4)	Distal metacarpal proximal and middle phalangeal fractures (F and FC), MCPJ & PIPJ injury (L)	Four–six weeks; non/postoperatively

Dynamic finger orthoses (Figure 4)	Combined with *buddy-taping* of two adjacent fingers	Proximal phalangeal fractures (F): criteria for nonoperative treatment (see the paper)	Four–six weeks; *double-finger stall* splinting principle; early functional treatment in cast

F: fracture treatment; FC: fracture treatment children; L: luxation/ligament injury; D: degenerative connective tissue disease; for other abbreviations, see the paper.
